# Long-term outcome of irradiation with or without chemotherapy for esophageal squamous cell carcinoma: a final report on a prospective trial

**DOI:** 10.1186/1748-717X-7-142

**Published:** 2012-08-22

**Authors:** Mina Liu, Xuehui Shi, Xiaomao Guo, Weiqiang Yao, Yong Liu, Kuaile Zhao, Guo-Liang Jiang

**Affiliations:** 1 Department of Radiation Oncology, Fudan University Shanghai Cancer Center; Department of Oncology, Shanghai Medical College, Fudan University, Shanghai 200032, China

**Keywords:** Esophageal squamous cell carcinoma, Chemoradiation, Long-term follow-up

## Abstract

**Purpose:**

To investigate the long-term outcome of esophageal squamous cell carcinoma (SCC) treated by irradiation with or without concurrent chemotherapy.

**Methods and materials:**

A prospective clinical trial was carried out from 1998 to 2000. One hundred and eleven patients were randomly enrolled to receive either late course accelerated hyperfractionated irradiation (LCAF) or LCAF with concurrent chemotherapy (LCAF + CT). For LCAF, 41.4 Gy in 23 fractions was first delivered at five fractions per week, followed by 27 Gy in 18 fractions at two 1.5 Gy fractions a day. Concurrent chemotherapy of cis-platinum and 5-fluorouracil was administered for four cycles. Overall survival (OS), locoregional recurrence and distant metastasis were observed. Late toxicity was scored by RTOG criteria, and quality of life (QOL) was also evaluated.

**Results:**

The median follow-up time was 24 months for all patients and 138 months for 17 living patients. Median survival time was 25 months and 32 months in LCAF and LCAF + CT (*p* = 0.653), respectively. For an entire group of patients, overall survivals were 34%, 27% and 22%; locoregional recurrence rates were 30%, 36% and 41%; and distant metastasis rates were 26%, 28% and 29% at 5-yr, 8-yr and 10-yr, respectively. Incidences of ≥ Grade 3 late toxicity were 29% at 10-yr. There were no statistically significant differences between LCAF and LCAF + CT with respect to the parameters mentioned above. Cumulative incidence of late toxicities of ≥ Grade 3 increased sharply after the attained age of 70 years. Eighty-eight percent of patients lived with good KPS (≥ 90) and 94% could eat regular or soft diet.

**Conclusion:**

The long-term outcome of esophageal SCC patients who received LCAF or LCAF + CT was good. The locoregional and distant failures occurred more often in the first three years after treatment, but could continuously occur up to 10 years. The late toxicity was acceptable. Late toxicities ≥ Grade 3 were more likely to occur in elderly patients. QOL was good in living patients.

## Introduction

Esophageal carcinoma is one of the most common cancers in the world with an estimated 482,300 new cases and 406,800 yearly deaths worldwide [1]. Currently, concurrent chemoradiation is the standard of care for esophageal carcinoma. However, outcome is still unsatisfactory due to poor local control. To improve local controls, unconventional fractionated irradiation for esophageal carcinoma has been investigated since the 1990s. In our center, we started the clinical trials on late course accelerated hyperfractionated irradiation (LCAF) in 1988 [2]. We hypothesized that tumors began accelerated proliferation around 4 weeks after the commencement of irradiation. Thus, we used accelerated irradiation about 4.6 weeks after conventional fractionation irradiation in the hopes that LCAF could effectively inhibit the accelerated proliferation of surviving tumor cells. In the 2000s, we reported a 5-year overall survival rate of around 30% [3-6]. In order to further improve the treatment efficacy, we combined chemotherapy concurrently with LCAF (LCAF + CT) in a prospective randomized clinical trial, which demonstrated a trend towards better survival compared to LCAF alone [3]. In the current article, we presented the long-term outcome of this trial after a 10 year follow-up, which includes survival, failure patterns, late toxicity and quality of life (QOL).

## Methods and materials

### Study design

From March 1998 to July 2000, 111 patients were recruited into a prospective randomized clinical trial. The patient eligibility criteria were as follows: (1) Esophageal squamous cell carcinoma (SCC) confirmed by histology or cytology; (2) Clinical stages of T_1-4_ N_0-1_ M_0_ (UICC, 1997); (3) Baseline of laboratory tests met chemoradiation requirements, including a white blood cell count of >  4.0*10^9^/L, platelet count of > 100 *10^9^/L, adequate renal function (serum creatinine concentration of < 1.5 mg/dL, blood urea nitrogen of < 8 mmol/L and creatinine clearance of > 65 mL per minute), and normal liver function; (4) Karnofsky performance status (KPS) ≥ 70; (5) No prior therapy; (6) No previous malignancies; and (7) No serious comorbidity that would preclude safe administration of treatment. Exclusion criteria included the followings: (1) Evidence of esophageal perforation or deep ulceration; (2) Complete obstruction of the esophageal lumen; (3) Esophageal bleeding; (4) Involvement of supraclavicular lymph nodes; and (5) Distant metastases. Pretreatment evaluations for staging included chest computed tomography (CT), esophageal barium photography and ultrasound examinations of liver, kidney, spleen and retroperitoneal lymph nodes.

Patients were randomized into two arms, LCAF + CT or LCAF alone. Informed consent was obtained from each patient on the day of admission. The study protocol conforms the guidelines of the World Medical Association. Declaration of Helsinki. The ethic’s Committee of our approved the protocol and all patients gave their informed consent for the inclusion in the study.

### Treatment arms

#### LCAF

This consisted of two phases. In the first phase, 41.4 Gy in 23 fractions was delivered by conventional fractionation of 1.8 Gy per fraction, one fraction per day and five fractions per week. In the second phase, 27 Gy in 18 fractions was given by two 1.5 Gy fractions per day, each with an interval of > 6 h. A total of 68.4 Gy was irradiated in 41 fractions for 6.4 weeks.

Irradiation was carried out with a 6-MV photon and a two anterior oblique field technique associated with a pair of appropriate wedges for cervical esophageal lesions or a three-field approach with one anterior and a pair of posterior oblique fields for lesions in the thorax. Irradiation portals covered the primary tumor and metastatic nodes, which were shown on CT and a barium photographs. Margins of 2–3 cm were added for subclinical invasion and set-up error. At the long axis, margins of 3 cm proximally and 5 cm distally were set to sufficiently encompass the submucosal invasion. The dose was prescribed to the isocenter without inhomogeneity correction. In the second phase of irradiation, the irradiation fields were reduced to 2-cm margins beyond the superior and inferior ends of the esophageal lesion. No prophylactic irradiation was given in the supraclavicular regions.

#### Chemotherapy

In the LCAF + CT arm, patients received concurrent chemotherapy once a month for four cycles of PF regimen (cis-platinum of 25 mg/m^2^ daily and 5-Fluorouracil of 600 mg/m^2^ daily for three consecutive days) during and after irradiation.

### Follow-up

Follow-up was performed on patients every four months for the first year, every six months for two years, annually for two years, and every other year, thereafter. Each follow-up visit included complete history, physical examination, evaluations of quality of life, a blood test, a chest X-ray film, esophageal barium radiography and a chest CT. Late treatment-related toxicity was scored by RTOG criteria.

### Definition of locoregional recurrence

Locoregional recurrence was defined as esophageal and/or regional lymph node failures. Once esophageal recurrence was suspected, a biopsy was required. When node recurrence was suspected, CT, MRI, or PET/CT should be performed. Lymph node recurrence was determined by one of the following criteria: (1). Nodes reappeared after complete disappearance; (2). Nodes were enlarged after remaining stable; and (3). New nodes of > 1 cm in diameter appeared in the mediastinal or abdominal regions where no enlarged nodes existed prior to irradiation.

### Distant node metastasis

Distant node metastasis included M1a and M1b (UICC, 2002). M1a was referred to as supraclavicular or cervical lymph node metastases from the upper thoracic esophageal SCC or celiac axis nodes from the distal esophageal. M1b included all other patterns of distant nodal spread.

### QOL assessment

QOL was evaluated by the following criteria: (1) The diet that patient can swallow, which was categorized into regular diet, soft food, semi-fluid food and fluid food; (2) Cough, which was graded according to common terminology criteria for adverse events (CTCAE, version 4.0); (3) Hemoptysis by CTCAE (version 4); and (4) General conditions by KPS score.

### Statistical analysis

Overall survival rate (OS), locoregional recurrence rate, and distant metastasis rate were the main endpoints estimated by the Kaplan-Meier model (KM) (SPSS, version 16.0) and the significance of difference was evaluated by the log-rank test. The incidence of severe late toxicity, which was defined as those of Grade ≥ 3, was also estimated by KM model. Attained age was used, which was defined as the age when events occurred (Attained Age = Age at diagnosis + Time to the event). The observation started from the first day of treatment until death or the last follow-up visit.

## Results

### Survival

From March 1998 to July 2000, 111 patients were randomly enrolled in LCAF (57) and in LCAF + CT (54). The clinical characteristics were well balanced between the two arms (Table 1). Ninety-four patients died by the last follow-up visit in December 2010 and 17 patients survived with 9 patients in LCAF and 8 patients in LCAF + CT. The median follow-up time was 24 months (1 month to 128 months) for entire group and 138 months (126 months to 152 months) for those alive.

**Table 1:** Patient clinical characteristics

	**LCAF**	**LCAF + CT**	**P value**
No. of patients	57	54	
Gender, n (%)			
Male	36 (63)	42 (78)	0.092
Female	21 (39)	12 (22)	
Age (yr)			
Median (range)	61.0 (41–74)	54.5 (39–74)	0.433
KPS, n (%)			
70	3 (5)	2 (4)	0.692
80-100	54 (95)	52 (96)	
Lesion location, n (%)			
Cervical	3 (5)	4 (7)	0.724
Upper thorax	18 (32)	12 (22)	
Middle thorax	34 (60)	36 (67)	
Lower thorax	2 (3)	2 (4)	
Esophageal length at long axis			
Median (range) (cm)	6.0 (1–10)	6.0 (2–9)	0.132
Stage, n (%)			
T1-2N0M0	11 (19)	11 (20)	0.690
T3-4N0M0	37 (65)	37 (69)	
T1-4N1M0	9 (16)	6 (11)	

**Figure 1:**
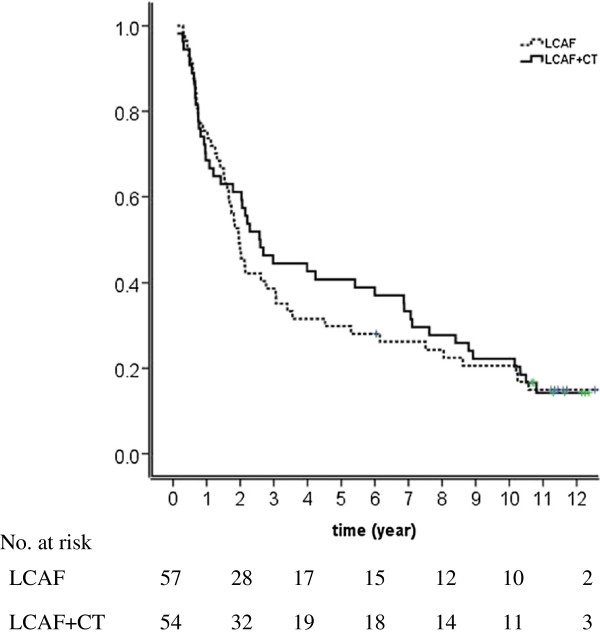
Overall survival in patients treated with late course of accelerated hyperfractionated irradiation (LCAF) alone (dashed line) or LCAF with concurrent chemotherapy (LCAF + CT) (solid line).

Median survival times were 25 months (CI, 21.3-28.7) and 32 months (CI, 8.6-55.4) for LCAF and LCAF + CT, respectively. OS at 5-year, 8-year and 10-year were 34%, 27% and 22% for entire group. There were no significant differences between LCAF and LCAF + CT arms in 5-year (28% vs 40%), 8-year (21% vs 29%) and 10-year (19% vs 23%) (Figure 1, p = 0.653).

**Figure 2:**
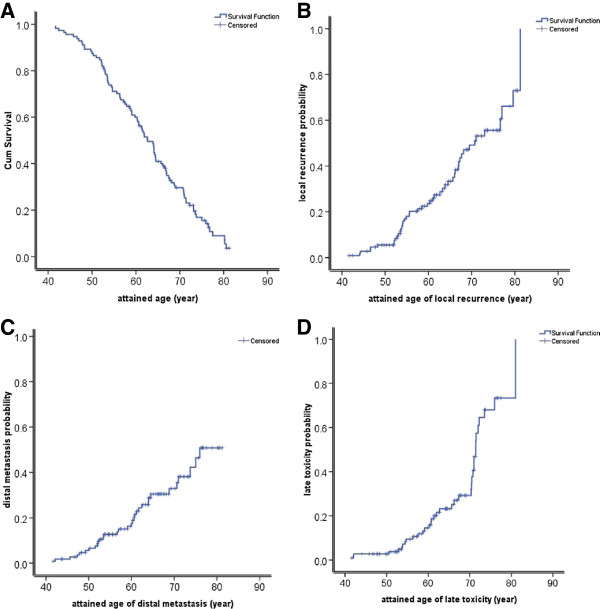
Overall survival (A), locoregional recurrence rates (B), distant metastasis rates (C) and late toxicity incidences (D) on attained age for entire group of 111 esophageal squamous cell carcinomas.

From a univariate analysis, clinical stage and tumor length at the long axis were demonstrated significant prognostic factors for OS (Table 2). Overall survival on attained age is illustrated in Figure 2-A.

**Table 2:** Prognostic factors for overall survival in esophageal squamous cell carcinoma treated by late course of accelerated hyperfractionated irradiation (LCAF) or late course of accelerated hyperfractionated irradiation with concurrent chemotherapy (LCAF + CT)

**Parameter**	**P value**
Age (≥ 70 yr vs. < 70 yr)	0.221
Stage (I vs. II + III)	0.047
KPS (≥ 90 vs. < 90)	0.633
Length (< 7 cm vs. ≥ 7 cm)	0.021
Treatment (LCAF vs. LCAF + CT)	0.653

### Pattern of failures

#### Locoreginal failure

Locoregional failures were documented in 46 patients, of whom 37 patients had recurrence in the esophagus, 6 patients in mediastinal nodes and both esophagus and mediastinal nodes in 3 patients. Among locoregional recurrence patients, 12 were diagnosed by biopsy and the rest by CT and/or PET or endoscopy, but no biopsy was performed either because of the patient’s refusal or due to an esophageal lumen that was totally obstructed, thus precluding biopsy.

Of the 46 locoregional failures, 42 were in-field recurrences, which included 38 in in-boost dose region; 1, out-boost dose region; and 3, both in- and out-boost dose regions. Three patients experienced relapses in out-field mediastinal lymph nodes. The last patients developed a second primary cardia carcinoma 22 months after treatment.

Thirteen patients with locoregional recurrence received salvage therapy including surgery (7), irradiation (4) and chemoradiation (2). The remaining 33 patients received no further therapy. Finally, four patients were salvaged and 42 died of locoregional recurrences or distant metastases.

**Figure 3:**
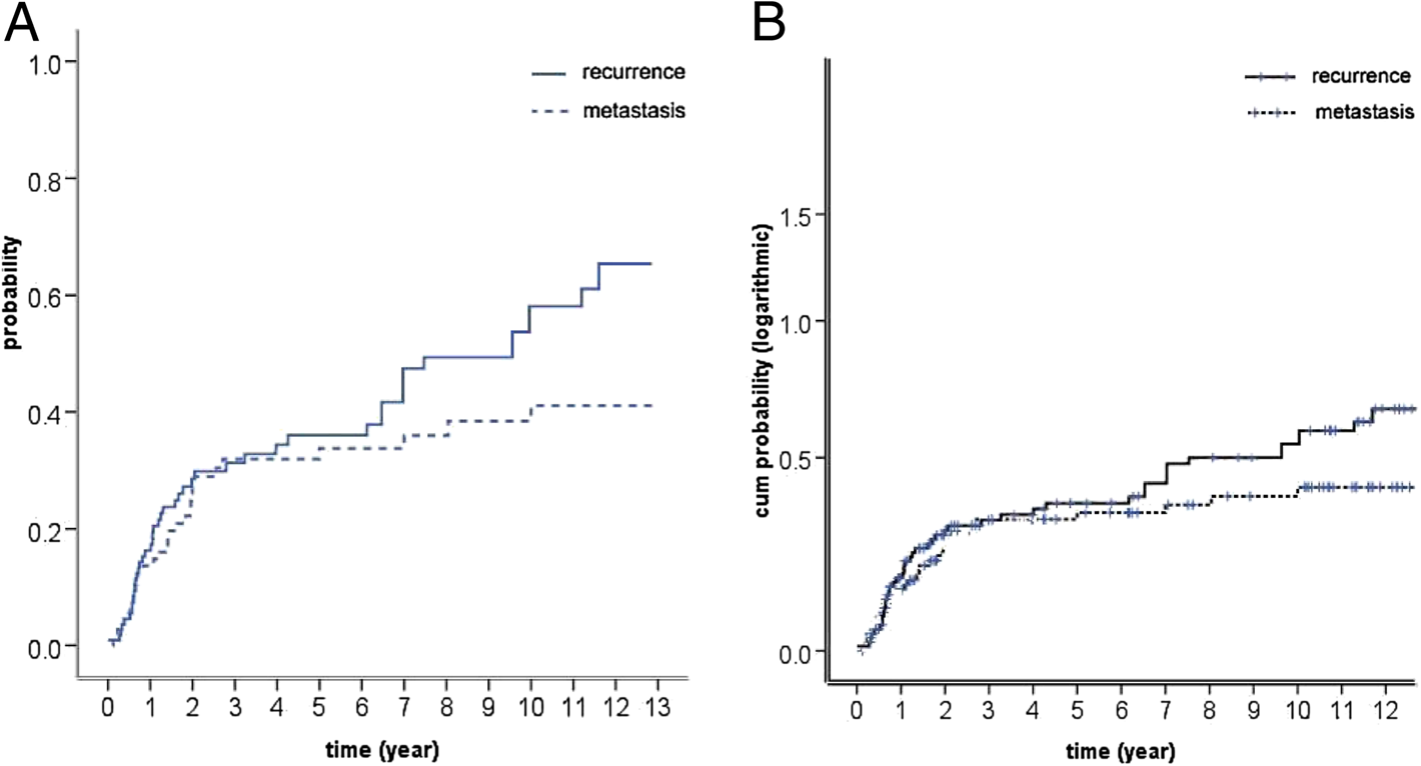
**Locoregional recurrence (solid line) and distant metastasis (dashed line) rates for entire group of 111 esophageal squamous cell carcinomas.A**: The probability was plotted on linear scale; **B**: The probability was plotted on logarithmic scale.

**Figure 4:**
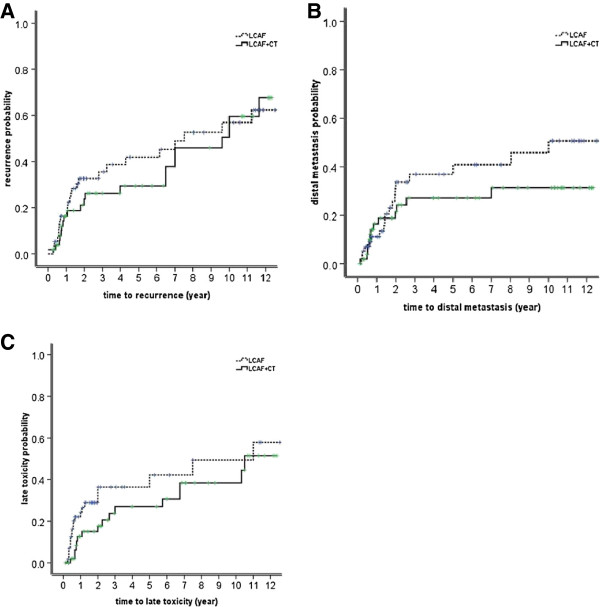
Locoregional recurrence (A), distant metastasis (B) and late toxicity (C) in esophageal squamous cell carcinoma treated by late course of accelerated hyperfractionation accelerated irradiation (LCAF) (dashed line) or LCAF concurrent chemotherapy (LCAF + CT) (solid line).

The locoregional recurrence rates are shown in Figures 3 and 2-B and they were relatively constant after three years. The locoregional recurrence occurred more often in the first two to three years and then increased steadily at a low rate for up to 13 years after treatments. For all 111 patients, the 5-year, 8-year and 10-year locoregional recurrence rates were 30%, 36% and 41% respectively. For LCAF, these rates were 35%, 40% and 44% and for LCAF + CT they were 25%, 32% and 40% respectively (*p* = 0.558) (Figure 4-A).

#### Distant metastasis

Distant metastases developed in 32 patients, including distant node disease (11), lungs (11), bone (3), brain (2), liver (1), stomach (1) and multiple organs (3). Distant metastasis rates are illustrated in Figures 3 and 2-C. The majority of distant failures occurred three years after therapy and hazard rate (HR) was constant after three years. For the entire group, the 5-year, 8-year, and 10-year distant metastasis rates were 26%, 28% and 29% respectively. There was no significant difference between the two arms, namely 5-year, 8-year, and 10-year distant metastasis rates of 30%, 32% and 33% for LCAF, respectively, and 23%, 25% and 25% for LCAF + CT, respectively (*p* = 0.264) (Figure 4-B).

### Late toxicity

Grade 3 or higher late toxicities occurred in 32 patients (29%), including pulmonary fibrosis in 21 patients (19%), esophageal stenosis in 10 patients (9%) and pericarditis in 1 patient (1%). Five out of these 32 patients died of pulmonary or heart failures due to the severe pulmonary fibrosis. The cumulative late toxicity incidences at 5-years, 8-years and 10-years were 26%, 29% and 29%, respectively. There was no significant difference between the two arms, namely 30%, 33% and 33% for LCAF and 21%, 26% and 26% for LCAF + CT (*p* = 0.161) (Figure 4-C).

The incidence of late toxicities of Grade 3 or greater on attained age is also displayed in Figure 2-D. At the attained age of 50-year, cumulative probability of late toxicities was less than 10% and it rose to about 30% at the attained age of 70-years. However, at 80-years it rose to 70%.

Five patients (three patients in LCAF and two patients in LCAF + CT) succumbed to intercurrent diseases. Two of the deaths resulted from massive hemoptysis at eight months and 14 months after treatment. One patient had a lethal pulmonary infection, which led to respiratory failure six months after therapy. One patient died of pulmonary infarction 31 months after treatment. The last patient who did not have any history of cardiovascular diseases died of a heart attack at the age of 60, six years after treatment.

One patient developed a second primary cancer, leiomyosarcoma, four years after therapy, located in the irradiated segment of esophagus. This patient received surgery and was doing well up until the last follow-up visit.

### QOL

QOL was not evaluated periodically or well documented due to over 10 years of follow-up, which made the data disintegrated. Therefore, here, we presented QOL data on 17 living patients at the last follow-up with median follow-up time of 138 months (Table 3). Fifteen of patients (88%) lived with good KPS (≥ 90). Thirteen patients (76%) could eat regular food. Cough and hemoptysis occurred at Grade 1 in 24% and 6% of the patients, respectively.

**Table 3:** Quality of life evaluation in 17 patients alive at the last follow-up visit

**QOL**		**No. of patients (%)**
KPS	≥ 90	15 (88%)
	70-80	2 (12%)
Diet	Regular	13 (76%)
	Soft food	3 (18%)
	Semi-fluid	1 (6%)
Cough	No	13 (76%)
	Grade 1	4 (24%)
Hemoptysis	No	16 (94%)
	Grade 1	1 (6%)

## Discussion

We reported that the long-term outcome of our clinical trial on esophageal SCC treated by LCAF alone or LCAF with concurrent chemotherapy after a 10-year follow-up period. For esophageal carcinoma, the concurrent chemoradiation has been recognized as the standard of care. However, the long-term outcome for failure patterns and very late treatment related toxicity has never been reported. Therefore, our study could be of significant interest to oncologists who are involved in the treatment of esophageal cancers.

### Survival

Five-year, 8-year and 10-year overall survival rates in our study were 34%, 27% and 22% respectively, which were comparable to the rates in concurrent chemoradiation in RTOG 8501 [7]. However, no significant difference was noticed between patients in LCAF alone and LCAF + CT, despite a trend of higher survival in chemoradiation around five years after treatment.

### Failure patterns

Findings observed in the failure patterns were interesting. It was obvious that locoregional failures continuously occurred up to ten years after treatment, although more often in the first three years. However, distant metastasis developed mostly in the first three years, and thereafter, increased slowly up to ten years. This result suggested that in the first three years, both locoregional and distant failures were major problems, but locoregional failures for long-term survivors should not be disregarded. For long-term survivors of esophageal SCC, the biological behaviors of the tumors were probably less malignant than that of the others, and thus less distant metastases occurred.

### Toxicity

We have already reported the acute toxicity in our previous publication, including severe (≥ Grade 3) acute radiation induced lung disease in 24 patients (22%) and severe (≥ Grade 3) esophagitis in 5 patients (5%) [3].

Late complications of Grade ≥ 3 were recorded in 32 patients (29%), mainly in the first three years after treatment. However, it occurred continuously for over ten years. The predominant late complications were pulmonary fibrosis and esophageal stenosis, which finally resulted in 16% (5/32) mortality. There were 29% of patients with late complications in our study, which was comparable to those reported by previous studies (28%-46%) [7-11]; despite the higher tumor dose in this study than those in RTOG 8501. It could be attributed to the lack of prophylactic irradiation to mediastinal, supraclavicular and celiac nodes and a reduced irradiation field after 41.4 Gy. Furthermore, the use of hyperfractionation further reduced the irradiation-induced toxicity.

The interesting finding from this study is the cumulative incidences of late complications (Figure 2-D). From age 40 to 50 years, the late toxicity incidence appeared to be relatively low (< 5%). From 50 years to 70 years, it accumulated at a more rapid rate to approximately 30%. After 70 years, it rose remarkably to over 70%. This showed that the prevalence of late toxicities was a manifestation for elderly patients. This finding is consistent with recent publications on pelvic cancers after combined therapy. The results from the Medical Research Council RT01 Trial showed that age was associated with late rectal bleeding and late proctitis in prostate cancer [12]. In a Swedish study of 1147 patients with rectal cancer, it was also evident that older patients (aged 75 years and above) were at greater risk of late gastrointestinal bleeding (RR = 3.81) [13]. Therefore, the late toxicity induced by treatments was predominantly a problem that occurred in the elderly population. Therefore, considering that late toxicities of Grade 3 or greater were more likely to occur in elderly patients with esophageal SCC, we proposed that chemoradiation should be cautiously delivered to elderly patients due to their higher risk for late toxicity.

Five patients died of intercurrent diseases with neither ≥ Grade 3 late complications nor disease recurrences; thus, we believe that their deaths could be partly attributed to irradiation or chemotherapy. One patient died of pulmonary infection, which could have resulted from jeopardized immune response after treatment. An invasion of great vessels could be one of the reasons two patients died of massive hemoptysis. The other two patients who died of pulmonary infarction and heart attack likely had injuries of blood vessels by irradiation as contributing factor to their death. Another possibility might be that radiation-induced injury of vessels worsened their pre-existed vascular diseases. The exact irradiation doses to heart and blood vessels were, however, unknown because of the lack of 3-dimensional dose distributions. A second primary tumor of esophageal leiomyosarcoma could also be related to chemoradiation. Therefore, for long-term survivors, we should also be aware of the risk of treatment-induced secondary primary malignancies.

### QOL

For 17 living patients, after a median follow-up time of 138 months, their QOL was quite good. A majority of the patients lived with good performance status and could eat regular food with no severe treatment-related sequences. In the current study, we used a two-dimensional irradiation technique, whereas the late complications were acceptable. If modern irradiation techniques of three-dimensional conformal radiation therapy or intensity modulated radiation therapy were used, the late toxicity could be further reduced.

In spite of our effort, the outcome was still not satisfactory for esophageal SCC patients. A prospective clinical trial on concurrent use of chemoradiation and nimotuzumab, a monoclonal antibody against epidermal growth factor receptor, is underway in our center in an effort to further improve local control and survival for esophageal SCC patients [14].

In conclusion, the local control and survival of LCAF irradiation with or without concurrent chemotherapy was good after a 10-year follow-up for esophageal SCC. The locoregional failure occurred gradually up to 10 years after treatment, as did distant metastasis, although with a higher rate in the first three years. The late complications were acceptable and patients’ QOL was good. However, late treatment toxicities were more likely to occur in elderly patients. Therefore, chemoradiation should be cautiously delivered to the elderly patients due to their higher risk for late toxicity.

## Competing interests

The authors declare that they have competing interests.

## Authors’ contribution

Each author had participated sufficient in the word. XH Shi, Zhao and JG Liang designed the research. MN Liu, XM Guo, WQ Yao and Y Liu collected the data and performed the statistical analysis. Finally, the manuscript was written by MN Liu. All other authors helped. All authors read and approved the final manuscript.
